# Feasibility of an Intervention for Patients with Cognitive Impairment Using an Interactive Digital Calendar with Mobile Phone Reminders (RemindMe) to Improve the Performance of Activities in Everyday Life

**DOI:** 10.3390/ijerph17072222

**Published:** 2020-03-26

**Authors:** Maria Andreassen, Helena Hemmingsson, Inga-Lill Boman, Henrik Danielsson, Tiny Jaarsma

**Affiliations:** 1 Department of Health, Medicine and Caring Sciences, Linköping University, 601 74 Norrköping, Sweden; helena.hemmingsson@specped.su.se (H.H.); tiny.jaarsma@liu.se (T.J.); 2 Department of Special Education, Stockholm University, 106 91 Stockholm, Sweden; 3 Department of Rehabilitation Medicine, Danderyd University Hospital, 182 88 Stockholm, Sweden; il_boman@hotmail.com; 4 The Swedish Institute for Disability Research and The Department of Behavioural Sciences and Learning, Linköping University, 581 83 Linköping, Sweden; henrik.danielsson@liu.se

**Keywords:** acquired brain injury, assistive technology, occupational therapy, rehabilitation, smartphone, stroke

## Abstract

The aim of this study is to increase evidence-based interventions by investigating the feasibility of an intervention using an interactive digital calendar with mobile phone reminders (RemindMe) as support in everyday life. Qualitative and quantitative data were collected from participating patients (*n* = 8) and occupational therapists (*n* = 7) from three rehabilitation clinics in Sweden. The intervention consisted of delivering the interactive digital calendar RemindMe, receiving an individualized introduction, a written manual, and individual weekly conversations for two months with follow-up assessments after two and four months. Feasibility areas of acceptability, demand, implementation, practicality, and integration were examined. Patients expressed their interest and intention to use RemindMe and reported a need for reminders and individualized support. By using reminders in activities in everyday life their autonomy was supported. The study also demonstrated the importance of confirming reminders and the possible role of habit-forming. Occupational therapists perceived the intervention to be useful at the rehabilitation clinics and the weekly support conversations enabled successful implementation. This study confirmed the importance of basing and tailoring the intervention to patients’ needs and thus being person-centered.

## 1. Introduction

Cognitive impairment (CI) due to stroke, traumatic brain injury (TBI), or another neurological disease can result in difficulties for people to take the initiative and to plan, structure, and organize everyday life as well as to remember to carry out planned activities [1,2]. This can result in an experience for patients of lacking control over everyday life or a sense of being dependent on family and friends, and this can have an impact on their perceived quality of life [1,3]. Enhanced occupational performance can increase a person’s perception of the quality of life, self-efficacy, and participation [4]. Digital support can increase the perceived quality of life and sense of independence for people with CI [3,5].

New technologies such as smartphones have the potential to support people with CI to perform activities in everyday life [6] in comparison with traditional compensatory strategies (such as paper-calendar), and digital support can provide reminders via alarms and/or text messages [7]. Such tools include personal digital assistant device [8], tablets [9], smartphones with or without apps [6,10], smartwatches [11] or short text message service (SMS) to the mobile phone [12] A smartphone includes several of the functions that can serve as cognitive aids for adults with CI. However, research has shown that older people with CI who own smartphones use calendars or reminders to a lesser extent than younger adults [13]. Therefore, there is a need for user-friendly, flexible, and interactive digital calendars with active reminders to support activities in everyday life. RemindMe, an interactive web-based calendar, was developed and designed to support people with CI and includes three core functions: (i) scheduling of activities and customizing reminders in a user-friendly digital calendar; (ii) active confirmation of reminders sent by (SMS) requiring users to acknowledge the prompt actively by responding the SMS; and (iii) a feedback system which registers information based on the user’s interaction with RemindMe. It is optional for the user to invite a support person with access to the user’s account in RemindMe [14]. A novel feature with RemindMe is the feedback system, in which the user actively needs to reply to the SMS. The user can get feedback on whether an activity was performed or not, when a responding SMS was sent and an overview of the registered activities.

RemindMe has been tested for usability from the perspective of professionals supporting people with CI and was found to be useful, easy to use and intuitive [15]. RemindMe has also been used by community-dwelling seniors for 6 weeks who found the core functions to be beneficial, especially the active reminders and the opportunity to acknowledge the reminder by answering the SMS [14]. According to The National Board of Health and Welfare [16] and national clinical guidelines for stroke [17], there is clinical evidence that compensatory interventions with reminders work well for people with memory deficits after stroke. However, there is lack of research evidence of effective interventions that support the performance of everyday life activities [16].

Nowadays interventions in health care often use digital technology. However, there is a lack of studies that investigates elderly people’s ability to learn and use digital technology in real life [18] Additionally, the Medical Research Council advises that interventions should be tested thoroughly for feasibility before starting larger trials. Feasibility studies are needed to identify the crucial parts of the intervention, using both qualitative and quantitative research methods [19].

Feasibility studies have various designs that can support the researcher to plan an randomized controlled trial (RCT) study [20,21,22] by producing findings that help to determine whether or not an intervention should be recommended for further testing [20]. To ensure that an intervention will lead to the intended outcome it is important to conduct feasibility testing before a major study is conducted. This is especially important for complex interventions including several variables [19] and studies with vulnerable patients [9]. Interventions need to be tested within everyday practice to examine the possibility of performing the intervention and to find out the strengths and weaknesses of the planned intervention. To support designing feasibility studies Bowen et al. [20] have created a structure with eight areas of feasibility important to consider. The areas are acceptability, demand, implementation, practicality, adaptation, integration, expansion, and limited efficacy. Each area can be used to determine whether an intervention can work, does work, or will it work. The scope in this study, is to investigate whether an intervention with RemindMe ‘will work’ in real-life settings, by examine five of the areas suggested by Bowen et al. [20] (acceptability, demand, implementation, practicality, and integration). This is the first step before moving on to further test the effectiveness of the intervention for patients with CI. The areas of adaptation, expansion and limited efficacy were excluded as they do not support the aim of this study.

The aim of this study is to increase evidence-based interventions by investigating the feasibility of an intervention for patients with cognitive impairment using an interactive digital calendar with mobile phone reminders (RemindMe) as a support for the performance of activities in everyday life.

## 2. Materials and Methods

### 2.1. Study Design

This feasibility study used both qualitative and quantitative research methods as recommended by Bowen et al. [20], O’Cathain et al. [21], and Orsmond and Chon [22].

### 2.2. Participants and Recruitment

Eight patients and seven occupational therapists (OTs) from three rehabilitation clinics in Sweden (specialists in rehabilitation for people with neurological impairments in primary care) participated in the study.

The patients were outpatients and participated in an intervention group in a pilot study [23]. Patients were included if they had CI and were in need of support with planning, organizing and remembering to do activities in everyday life and if they had access to a computer/tablet and mobile phone/smartphone. Patients also needed to have the linguistic ability to participate in the data collection. Eight patients, six males, and two females, with a median age of 58 years (range 26–68) used RemindMe during the study period. The patients had CI due to stroke (4 out of 8), TBI (2 out of 8), sepsis (1 out of 8), and multiple sclerosis (1 out of 8). One patient had completed primary school as the highest educational level, six patients had secondary school education and one had higher than secondary school education. All patients were accustomed to using smartphones on a regular basis.

Seven OTs working at the three rehabilitation clinics had recruited patients and were invited to participate in face-to-face interviews about the feasibility of the intervention with RemindMe. All chose to participate in the interview study. All were female, had worked for several years since graduation, and were experienced in rehabilitation of patients with CI (median 20 years, range 2–25 years). Five had a bachelor’s degree in occupational therapy and two had a bachelor’s degree with 1 year’s postgraduate education. The occupational therapists had the experience of using digital technology for their patients. However, no one was specialized in developing information and communication technology.

### 2.3. Intervention

The intervention in this study was designed to support people in performing activities in everyday life by using RemindMe. All OTs received training in use of RemindMe together with a written manual. The patients received rehabilitation as usual at the rehabilitation clinic together with a structured support in use of RemindMe as a support for memory and/or to plan and structure activities in everyday life.

In the first part of the intervention, the patients were given access to RemindMe and a user profile was created (time requirement approximately 30 min). Training in the use of RemindMe was given (by the first author) to the patient and if required to family members. Patients were provided with a written manual. The patients chose activities, based on their experiences, which they would like to receive reminders of, chose time they would like to receive the reminder and patients received support with scheduling the calendar (time requirement approximately 60 min).

The next part of the intervention was the structured use of RemindMe which consisted of individual conversations with patients conducted once a week (time requirement approximately 15 min each) for 2 months by the OTs at the rehabilitation clinic or by a Research Assistant (RA) (who was not responsible for intervention delivery). At the weekly conversations, support was given about the use of RemindMe regarding reminders sent in the previous week and strategies to schedule the coming week, and reminders were set up. After 2 months, the patients decided whether they wanted to continue to use RemindMe, but the weekly conversations with the OT/RA stopped (time requirement in total for weekly conversations approximately 120 min).

The last part of the intervention involved follow-up sessions after 2 and 4 months (conducted by the first author) concerning patients’ use and future use of RemindMe (time requirement approximately 15 min each). The time requirement in total for the intervention with RemindMe is estimated at 4 h. In this study, the recruiting OTs and recruited patients had access to RemindMe without any cost up to 1 year after the study period.

### 2.4. Data Collection

Data collection was organized using the approach described by Bowen et al. [20] to investigate the feasibility of the RemindMe intervention. The feasibility area of acceptability examines patients’ and OTs’ opinions of the intervention, demand examines the actual use of the intervention activities, implementation examines experiences when the intervention is delivered and the required resources, practicality examines if the intervention can be delivered at the clinic and integration examines if the intervention can be integrated at the clinic. Table 1 gives a detailed description of methods for data collection and data analyses.

**Figure 1:**
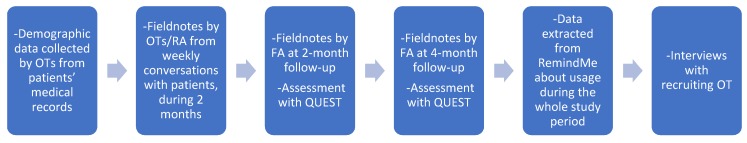
Flowchart describing the process of the data collection and used methods; OT—occupational therapist; RA—research assistant; FA—first author.

Qualitative data were collected by OTs/RA at the participating rehabilitation clinics to describe experiences about the intervention through descriptive field-notes and via face-to-face interviews with recruiting OTs by the first author. The field-notes were taken by OTs/RA from the weekly conversations during the first 2 months. Field-notes of patients’ perceptions of RemindMe were also taken at the follow-up session after 2 and 4 months by the first author. Face-to-face interviews with recruiting OTs were conducted after the intervention period, by the first author, with a semi-structured interview guide developed for this study. The guide included questions to explore the OTs’ perception of the intervention and about RemindMe as a digital support for patients with CI. An example of an interview question is: “Tell me about your perception of RemindMe as a product for patients with CI” followed by probing questions. All interviews were audiotaped and lasted between 15 and 30 min (median 23 min). See Figure 1 for a flowchart describing the various stages of data collection.

Quantitative data were collected to describe patients’ characteristics, the use of RemindMe and patients’ satisfaction with RemindMe. Demographic data were collected by the OTs at the rehabilitation clinic from the medical records. Data were also generated on an ongoing basis and were extracted from RemindMe. The data generated by RemindMe display activities that the reminders were used for, how often the activities occurred and the response rate of SMSs. Patients’ satisfaction with RemindMe was assessed with a self-report questionnaire, Quebec User Evaluation of Satisfaction with Assistive Technology 2.0 (QUEST) [24] after 2 and 4 months. QUEST 2.0 consists of two sections. Section one measures a person’s satisfaction with assistive technology and includes eight questions. Section two measures the perception of the received service when the assistive technology is delivered and includes four questions. Every question is rated on a five-point Likert-type scale from “1 = not satisfied at all” to “5 = very satisfied”. QUEST 2.0 has have been tested and found to have good reliability and validity [24]. The various steps of data collection are described in Figure 1.

### 2.5. Analyses

Qualitative data from field-notes and interviews were analyzed with content analyses [25]. Directed deductive content analyses are used when there is an existing model describing the examined phenomenon [25], and in this study was used to analyze the field-notes describing patients’ perceptions and the interviews with OTs. (Table 1). In this study, the feasibility of the intervention with RemindMe was studied according to the description of various areas and aspects of feasibility to consider by Bowen at al. [20]. The first author made a protocol of the field-notes and transcribed the interviews verbatim. To get a sense of the whole, the transcripts were read several times by the first author. The feasibility areas—acceptability, demand, implementation, practicality, and integration—were used as predetermined concepts for coding the text. Coding of the transcripts from the field-notes and interviews was done by highlighting text describing the feasibility areas. Text describing the five areas were made into categories. According to Bowen et al. [20], each feasibility area consists of various aspects. In the next step of the coding, text describing the feasibility aspects were highlighted and made into subcategories. Throughout the analyses, there was a back and forth movement between the transcriptions, the predetermined feasibility areas, and aspects of feasibility and the coding. To ensure the trustworthiness of the analyses the categories and subcategories were discussed by MA, HH, and TJ throughout the analyses.

Quantitative data of patients and OT demographic, descriptions from the self-report questionnaire QUEST 2.0 [24], plus data extracted from the web-based calendar to describe how patients used RemindMe were analyzed with descriptive statistics (frequencies, range, median) [26], see Table 1.

### 2.6. Ethics

This study was conducted in accordance with the Declaration of Helsinki and the project received ethical approval from the Regional Ethical Review Board in Linköping (D.nr 2016/145-31 and 2018/263-32). Patients and OTs received written and verbal information about the aim of the study before giving written informed consent to participate. Participating in the study was voluntary and participants could withdraw at any time without the need for explanation. The patients were informed that a support person/research personnel had access to the calendar for as long as the participant agreed.

## 3. Results

The results are presented and structured according to five of Bowen et al.’s [20] descriptions of areas necessary to consider when conducting a feasibility study, see Table 1. The five areas are acceptability, demand, implementation, practicality, and integration.

### 3.1. Acceptability

#### 3.1.1. Perceived Appropriateness

OTs perceived that it was appropriate to have a reminder via the mobile-/smartphone as most people keep their phones with them. It was also viewed positively that the user did not have to be skilled in using digital technology and that the reminder message was instructive, as described by one OT:

“The great thing about RemindMe is that you receive the reminder by SMS, and it says what to do, that is the advantage I think”

(OT 4)

The procedure to support learning and using RemindMe, included individual instruction, a written manual, weekly conversations and assessment after 2 and 4 months. The OTs perceived that the weekly conversation with patients was good and they could capture success or failure with the use of RemindMe in everyday life. It was reported that the procedure to support learning and using RemindMe helped patients to feel confident in using RemindMe, and some appreciated having the OT as a support person with insight in their personal calendar. Patients expressed that answering the SMS was an important support for them in remembering if an activity was performed, for example, if medication was taken or not. This indicates that the intervention with individual support for 2 months was acceptable according to patients’ needs and provided positive effects for the patients. One OT explained that by using RemindMe her patient had understood that he needed support and thus became interested in what kinds of support he would use.

“It was a gateway for him to understand that this was something that worked/… /later one he might try another kind of reminder system”

(OT 3)

However, it was pointed out that reminders already exist, and are integrated into smartphones nowadays, and since most people have smartphones, they can schedule reminders using an application within their smartphones. The calendar in RemindMe was designed to schedule from the computer, and the OTs perceived that this seemed difficult to maneuver. It was stated that it might be convenient if RemindMe included an application for a smartphone, as explained by one OT:

“I think it can be a bit tricky if you always have to schedule in the computer because that will be an extra step/…/ It would be neat if you can do the scheduling from the smartphone”

(OT 2)

However, it was also viewed that if a patient cannot handle a smartphone, they will have to use low technology and/or support from significant others instead.

#### 3.1.2. Satisfaction with RemindMe as a Product

Patients assessed their satisfaction with RemindMe using QUEST 2.0 [24] and the assessments indicated that patients were satisfied with RemindMe as a product, however, the range of satisfaction is wide. The patients were also satisfied with the service they got when RemindMe was introduced as well during its use, see Table 2.

### 3.2. Demand

#### 3.2.1. Expressed Interest or Intention to Use

Although patients experienced a need for reminders and expressed their interest in using RemindMe, some preferred to use the reminder system that they had used prior to the study and stated that RemindMe had no added value compared with the reminder system in their smartphone, or that they did not see the point in answering the SMS (patients 2,5). Other patients were initially eager to start and thus scheduled too many activities and needed support to reduce the number of reminders (patients 4,6). The patients expressed different needs: for some, it was to remember to do important activities, for example, taking medication/insulin, visit the rehabilitation clinic or other health care appointments (patients 1,2,4,8), while for others the most important need was to structure and support routines in everyday life (patients 3,6). Using the reminders seemed to support autonomy in activities in everyday life and positive aspects were that spouses did not have to remind them about these activities anymore (patients 1,4) and that reminders provided a good structure to know if insulin was taken or not (patient 4). A patient also expressed a feeling of security and comfort to know that reminders would come and thus activities would be executed (patient 3). Several patients expressed that it was difficult to maneuver and schedule the calendar from the smartphone (patients 2,5,6,8) and additional issues were related to remembering to adjust reminders due to daylight saving time (patients 1,7) or difficulties in scheduling when there was limited access to the internet (patient 7).

#### 3.2.2. Actual use of RemindMe

The patients’ actual use of RemindMe is described by data generated by RemindMe and shows activities for which the patients used RemindMe. The activities are grouped into two main areas:

- Pre-booked activities:
  ⚬ health care activity (seven out of eight patients) examples are visiting the rehabilitation clinic, visiting physician/dentist, attending medical assessments, and having a massage
  ⚬ leisure and exercise (six out of eight patients) examples are meeting family, friends, going on a weekend vacation, to a photography course, or a concert, and attending church; examples of exercises are visiting a gym or going golfing
  ⚬ errand (four out of eight patients) examples are meetings with authorities, leaving the car at a garage for repairs, and work-related activities

Everyday life activities:

⚬ daily routine (seven out of eight patients) examples are taking medication, undertaking an exercise program, watercolor painting, having lunch, watching TV, and making a daily phone call with a family member
⚬ schedule an appointment (five out of eight patients) examples are remembering to book an appointment with the authorities, reserving mobility services, and booking an exercise class

The feature of regular scheduling was used for some of the activities, for example, taking medication was scheduled every day, watching a TV-show every weekday or going to church on a specific weekday once a week. Other activities were scheduled incidentally.

Most patients used the feature of replying to the SMS, but the frequency varied a lot during the first 2 months (range 0–91%, median 56%). Three patients answered almost every SMS, three answered about half of the SMSs and two did not answer any of the SMSs. The use and response rates were the same during the following two months.

After the study period of four months, seven patients continued to use RemindMe (range 8–52 weeks, median 28 weeks). During this period, it differed a lot regarding whether patients used the function to answer reminder SMSs. Some patients did not answer at all, while others answered about half of the reminders (range 0–66%, median 13%), so the response rate is lower after the study period than during the study period. The fact that several patients chose to use RemindMe after the study period indicates that RemindMe fulfilled the need for reminders.

### 3.3. Implementation

#### 3.3.1. Success or Failure of Execution:

For successful implementation of RemindMe, the OTs expressed that they need to establish a thorough relationship with the patient and give support on a regular basis according to the patients’ individual habits and routines. This was particularly the case when scheduling, as was explained by one OT:

“It requires also that you have knowledge about the person/…/to ask the right questions/…/if the reminder needed to be sent half an hour ahead (of the activity)”

(OT 4)

Another aspect raised as necessary for successful implementation was the weekly conversation OTs had with patients because they gave support to the patients in everyday use, as one OT expressed:

“It seemed like the patient had more use of RemindMe when there was a regular evaluation every week about their usage”

(OT 5)

#### 3.3.2. Amount of Resources Needed for Implementation

In this study, there are no particular economic resources needed to implement RemindMe since patients do not have to pay for the use of RemindMe. But the system sends SMSs and they might incur a cost to answer the SMS (though not to receive it). However, several patients had telephone contracts with SMS costs included and thus incurred no cost for the use of RemindMe. However, one patient used a prepaid card with the smartphone so sending an SMS required payment. Patients also need to have access to the internet to be able to reach the web-based calendar for scheduling. Therefore, expensive mobile-/smartphone contracts or reduced access to the internet affected the implementation negatively.

### 3.4. Practicality

#### The Ability of Participants to Carry Out Intervention Activities:

The OTs pointed out some aspects that affected patients’ ability to carry out intervention activities. For some patients, it was difficult to carry out the needed intervention activities with RemindMe, especially if they had only used the smartphone to make phone calls or perceived the smartphone as difficult to handle.

“They need support to be able to use RemindMe, to get started, to practice, to schedule/…/ with CI, it can be difficult to set reminders yourself”

(OT 5)

It was necessary that patients were active in their everyday lives and thus experienced a need for reminders. OTs also expressed that patients’ prior habits and routines of using technology in their everyday life affected the ability to carry out intervention activities. This was related to the ability to learn since using new technology requires learning skills and can be very difficult for some patients.

### 3.5. Integration

#### Perceived Fit with Infrastructure

According to the OTs, the introduction period, evaluation by telephone, and responding, the reminders are aspects that could fit within the rehabilitation clinical setting. The introduction was perceived as a structure that could be used at the rehabilitation clinic when introducing digital support. The positive aspects were having a plan and structure of how to provide an introduction as well as doing evaluations on a weekly basis. It was also seen as an appropriate way to evaluate an intervention, especially when evaluation and scheduling could be done while talking to a patient on the telephone. Other positive aspects reported were that RemindMe could be used in treatment when planning, scheduling and evaluating rehabilitation activities, as explained by one OT:

“I could use RemindMe in treatment not only as a support for the patient in their everyday life. I could do the weekly schedule in RemindMe instead of doing it on paper. When I use paper, I don’t know if the patient has done the activity or not. Since they have memory impairment, it is difficult for them to tell”

(OT 4)

RemindMe might also be used as a support for patients in need of achieving a balance between activity and rest; for example, to schedule daily rest sessions and, by using the feedback system, to evaluate if the rest was taken or not.

“It would be awesome to use RemindMe and only schedule the rest and get feedback if rest was taken or not”

(OT 4)

It was also mentioned that RemindMe could be used in group treatments for patients with CI and in the group work with digital support for a few sessions. However, it was pointed out that complex interventions, with any kind of reminder, can become time-consuming as one intervention often reveals other problems as well. This was explained by one OT:

“We must have time. I had a patient with a spouse and children, and we tried a reminder system and it took a lot of time to include the whole family. It is a huge commitment”

(OT 1)

Another aspect affecting whether RemindMe would fit within a rehabilitation clinical setting was a consideration of rapid technological development and the fact that technology changes. Thus, according to OTs, it becomes vital to consider appropriate strategies to implement and use digital technology more than the technology itself. It is also a challenge for the OTs to stay updated with technological developments.

## 4. Discussion

This study investigated the feasibility of an intervention to support patients with CI in the performance of activities in everyday life and support the need for evidence-based interventions for patients with CI. The intervention consisted of RemindMe and a procedure to support learning and use of RemindMe. Results were analyzed with five areas from the framework for feasibility by Bowen et al. [20]. Overall, the interventions were found to be feasible, although some aspects of feasibility according to Bowen et al. [20] need to be considered.

Areas that supported feasibility were as follows: findings related to acceptability indicated that the intervention, which included individual support for two months, was perceived to be appropriate according to patients’ needs. Similar results, concerning intervention duration, was found in a study using SMS reminders in stroke rehabilitation [27]. Regarding the area of demand, patients expressed interest and their actual use of RemindMe showed that it was used to support activities in everyday life. Several patients chose to use RemindMe after the study period which indicates that RemindMe fulfilled their need for reminders. This is in line with other studies that highlight the potential of digital technology for people with CI, to compensate for lost ability [5,6,7,8]. Crucial for implementation was the individual support given on a regular basis. One example is to discuss strategies of how to incorporate digital support into daily routines [14]. Practicality aspects to consider were patients’ prior experience of and interest in technology, as well as prior habits and routines. According to the OTs, some parts of the intervention could be integrated into the rehabilitation clinic. Together, these data indicate that the intervention can be used in practice.

There were also areas that challenged feasibility. One major challenge found regarding acceptability, demand, and practicality was to do with navigating the calendar with the smartphone. This was mentioned both by patients and OTs. A smartphone-based application for scheduling might be a solution. An implementation aspect vital to consider is whether the patient has access to the internet at home or not and what kind of mobile-/smartphone contract the patient has as expensive contracts or reduced access to the internet affects the use of digital technology and thus affects implementation negatively. Another challenge for health care professionals within the area of integration is staying up to date with technological developments.

This feasibility study confirmed the importance of basing and tailoring an intervention to patients’ needs. In this study, we found that the intervention offers reminders that are based on patients’ perceived needs, for example, the individualized messages in reminder SMSs and the time for a reminder to be received. The personalized reminders together with the procedure to support use were all based on the patients’ needs and thus support the intervention to be person-centered. Research in other areas also shows that personal reminders are effective [28]. Data from RemindMe also confirmed that the patients used reminders for their everyday life activities. For person-centered practice, a thorough and respectful relationship between the therapist and the patients are needed, as are shared opinions about treatment [29]. When implementing interventions, it is needed to know patients’ experiences and interests, as well as prior habits and routines [4]; this was also found in a study of community-dwelling seniors who used RemindMe [14].

This study also demonstrated the importance of confirmatory reminders and the possible role of habit-forming [30]. It was vital to adjust the time and text in the reminder, so it arrived at the time of a specific situation and thus supported forming a new habit. To link an activity to a specific circumstance has been described by Fritz and Cutchin [30] as a procedure to establish new habits. Patients responded to the reminders in the first part of the study and taking medication was especially pointed out, as an activity where a responding reminder was appreciated. The action of confirmation of a reminder seems to support habit-forming. The present study highlights the advantage of a prompting reminder (“Remember to take morning medication”) being linked to an established everyday life habit (having breakfast) and therefore support to establish a new habit. In the later part of the study, the feature which encourages users to reply to the SMS was not used to the same extent. Several reasons are possible. One explanation could be that when a habit became established, the need for responding to the reminders is lower. However, it is essential to evaluate the user’s need for reminders on a long-term basis [6].

SMS messages have the potential to support the patient to take an active part in self-care activities, for example, to take medication [12]. In this feasibility study, the actual use of RemindMe showed that patients answered the SMS reminders and patients expressed that the opportunity to answer the reminder as well as to control the answer was important. Some patients expressed that the answer served as a confirmation and gave them information that the activity was done, and patients gained a sense of control and independence, and this reduced the need for support from spouses. This could at the same time result in reduce caregiver burden [9]. Spouses could feel safe to know an activity would become performed, without having to remind their partner of it.

Furthermore, this feasibility study showed that the procedure to support learning as well as giving usage support is vital when developing a product. The result from QUEST 2.0 [24] showed that the patients in this study were satisfied with both the service and the product at both time points. For service satisfaction, the median was five on the five-point scale for the first time point. This might indicate the importance of getting support when using new digital technology. In this intervention, “service” consisted of initial education, weekly conversations for two months (face to face at the rehabilitation clinic or by phone). This might support the learning process, as it gave support according to personal experiences on a long-term basis. Other important aspects were that the patients used their own smartphone which they were accustomed to. In a previous study, this was found as an aspect that supported community-dwelling elderly people to learn how to use digital technology [14]. Another positive aspect of using a mobile- or smartphone as support in daily life is that it is less stigmatizing when using the same digital technology as everyone else does [6].

The study gave insight into diverse situations that OTs experience in everyday practice. It was reported that RemindMe and the weekly conversations for two months, based on patients’ needs, were appropriate to support the use of reminders for everyday tasks since it gave a structured way to evaluate the rehabilitation interventions. A similar result was pointed out in a study using SMS reminders and weekly phone calls to evaluate progress in the rehabilitation of stroke patients [31]. Interventions spread out over a longer time period give patients time to practice and thus become more independent [29]. However, it was expressed that weekly phone calls during a longer period can become time-consuming and problematic to implement in daily practice. One concern was that the phone calls might reveal other, new, problems and therefore can frequent phone calls be problematic to implement in daily practice. As pointed out, another issue is the rapid technological development and the difficulty of keeping up to date. This is a major challenge for future healthcare practice. Ravenek et al. [32] highlight this issue and the need for health professionals to become skilled in matching the client’s need with the appropriateness of digital technology.

## 5. Strengths and Limitations

The strengths of this study were analyzing the intervention in a real-life setting together with the use of Bowen et al.’s [20] feasibility framework. RemindMe was used by patients in their everyday life and was integrated with treatment as usual at rehabilitation clinics to investigate if RemindMe was perceived as appropriate and if the intervention was possible to integrate, thus both patient and OTs’ perspectives were received.

One limitation of the study could be the small sample size with heterogenic diagnoses and ages. However, in a feasibility study, it is appropriate to have a small sample to determine whether the intervention is feasible or not before doing a major study [20].

Another limitation was the lack of a standardized conversation guide to support weekly conversations. When conducting a full RCT it would be recommended to develop a conversation guide so the weekly support conversations will become uniform and thus increase the reliability of field-notes covering patients’ reports of questions and other obstacles.

The feasibility areas of adaptation, expansion and limited efficacy [20] were not within the scope of this study, so it is unknown to what extent the intervention is feasible according to these areas. Further, the intervention’s effect on the caregiver burden was not evaluated in the present study. The perspective from the caregiver has been found to be an important matter to visualize [9] and needs to be further studied.

## 6. Conclusions

The results of this feasibility study are based on eight patients and seven OTs. From a patient perspective, the intervention with RemindMe seems to be feasible and supported patients to perform activities in everyday life. The OTs viewed the introduction procedure as structured and supportive but also time-consuming. To increase the body of knowledge and evidence-based interventions for patients with CI, further studies are needed to investigate the various effects of digital support on, for example, caregiver burden, independence, quality of life, or other psychosocial aspects.

## Figures and Tables

**Table 1:** Description of five of Bowen at al. [20] areas of focus for feasibility, the examined aspects of feasibility, data collection methods and methods for analyses.

Area of Focus for Feasibility	Aspects of Feasibility	Methods for Data Collection	Methods for Data Analyses
*Acceptability* To what extent the intervention is judged as suitable, satisfying or attractive to patients?	Perceived appropriateness	Data from interviews with OT about the intervention as appropriate to the patients	Directed deductive content analyses
	Satisfaction with RemindMe as a product	Assessed by patients with QUEST 2.0	Descriptive statistics (frequencies)
*Demand* To what extent the intervention is likely to be used?	Expressed interest or intention to use	Data from fieldnotes (by OT/RP, FA) about patients’ opinions of interest or intention	Descriptive statistics (frequencies)
	Actual use of RemindMe	Data of how patients used RemindMe (logs in the calendar)	Descriptive statistics (frequencies)
*Implementation* To what extent the intervention can be successfully delivered to the intended participants?	Success or failure of execution	Data from interviews with OTs about their perceptions of success or failure in implementing the intervention	Directed deductive content analyses
	Amount of resources needed for implementation	Data about the cost for the patients to use RemindMe	Data about RemindMe resources estimated by researchers’ experiences when participating in the study
*Practicality* To what extent can the intervention be carried out with intended participants using existing means, resources, and circumstances?	The ability of participants to carry out intervention activities	Data from interviews with OTs about patients’ ability to carry out intervention activities	Directed deductive content analyses
*Integration* To what extent the intervention can be integrated into an existing system?	Perceived fit with infrastructure	Data from interviews with OTs about their perceived fit of RemindMe within the infrastructure	Directed deductive content analyses

**Table 2:** Results from the assessment with QUEST 2.0 [24] to assess patient satisfaction with RemindMe as a product and satisfaction with the service, and the total QUEST-score of how satisfied patients were with RemindMe and the service.

Patients	Satisfaction with RemindMe		Satisfaction with Service		Total QUEST-Score, (Satisfaction with RemindMe and Service)	
	2 Months	4 Months	2 Months	4 Months	2 Months	4 Months
Patient 1	5	5	5	5	5	5
Patient 2	4.75	4.57	5	4.50	4.38	4.55
Patient 3	4.75	5	5	5	4.83	5
Patient 4	4.63	4.38	4.75	4	4.67	4.25
Patient 5	2.14	1.75	4	3	2.70	2.09
Patient 6	3.50	4.75	5	4.25	4	4.58
Patient 7	4.25	4.38	4.50	4.66	4.33	4.45
Patient 8	4.13	3.75	5	5	4.42	4.16
Median	4.44	4.48	5	4.58	4.40	4.50
